# Insanity

**Published:** 1887-11

**Authors:** 


INSANITY: A MANUAL FOR STUDENTS AND PRACTITIONERS. By E. C. Spitzka, M. D. Pp. 424. Price, $2.75. New York; E. B. Treat & Co., 1887.

Dr. Spitzka's reputation as a specialist in nervous diseases is such as to guarantee the accuracy and value of any work he may offer the profession. In this manual a vast amount of information on the causes and varieties of insanity is given. Part first gives nine chapters on classification and general characters of insanity; part second, in twenty-one chapters, treats of special forms of insanity; part third, seven chapters, considers the examination, management, etc., of the insane. While not an exhaustive treatise, this volume contains much information, and is very well worth its cost.

